# A new combination rule for Spatial Decision Support Systems for epidemiology

**DOI:** 10.1186/s12942-019-0187-7

**Published:** 2019-11-09

**Authors:** Luciana Moura Mendes de Lima, Laísa Ribeiro de Sá, Ana Flávia Uzeda dos Santos Macambira, Jordana de Almeida Nogueira, Rodrigo Pinheiro de Toledo Vianna, Ronei Marcos de Moraes

**Affiliations:** 1 Graduate Program in Decision Models and Health, Department of Statistics, Federal University of Paraíba, João Pessoa, Paraíba Brazil; 2 Department of Statistics, Federal University of Paraíba, João Pessoa, Paraíba Brazil; 3 Department of Nursing, Federal University of Paraíba, João Pessoa, Paraíba Brazil; 4 Department of Nutrition, Federal University of Paraíba, João Pessoa, Paraíba Brazil

**Keywords:** Epidemiology, Spatial analysis, Space–time analysis, Multiple Criteria Decision Making, Spatial Decision Support Systems, Brazil

## Abstract

**Background:**

Decision making in the health area usually involves several factors, options and data. In addition, it should take into account technological, social and spatial aspects, among others. Decision making methodologies need to address this set of information , and there is a small group of them with focus on epidemiological purposes, in particular Spatial Decision Support Systems (SDSS).

**Methods:**

Makes uses a Multiple Criteria Decision Making (MCDM) method as a combining rule of results from a set of SDSS, where each one of them analyzes specific aspects of a complex problem. Specifically, each geo-object of the geographic region is processed, according to its own spatial information, by an SDSS using spatial and non-spatial data, inferential statistics and spatial and spatio-temporal analysis, which are then grouped together by a fuzzy rule-based system that will produce a georeferenced map. This means that, each SDSS provides an initial evaluation for each variable of the problem. The results are combined by the weighted linear combination (WLC) as a criterion in a MCDM problem, producing a final decision map about the priority levels for fight against a disease. In fact, the WLC works as a combining rule for those initial evaluations in a weighted manner, more than a MCDM, i.e., it combines those initial evaluations in order to build the final decision map.

**Results:**

An example of using this new approach with real epidemiological data of tuberculosis in a Brazilian municipality is provided. As a result, the new approach provides a final map with four priority levels: “non-priority”, “non-priority tendency”, “priority tendency” and “priority”, for the fight against diseases.

**Conclusion:**

The new approach may help public managers in the planning and direction of health actions, in the reorganization of public services, especially with regard to their levels of priorities.

## Introduction

Decision making in a dynamic and rapidly evolving world is a great challenge, since several factors can influence the final decision, such as: the decision maker, conflicts of interest, the importance of the decision, different criteria involved in the problem, among others [1]. In the spatial context, the decision making process is also complex and requires spatialized information produced from many sources and interpreted by a variety of decision makers in relation to different criteria, objectives and/or alternatives [2].

A method that can take into account different criteria is the Multiple Criteria Decision Making (MCDM) defined as a set of procedures to help decision makers investigate multiple choice possibilities on the basis of multiple criteria and generate an order of preference for alternatives [3, 4].

The use of MCDM allows structuring the decision making process in well-defined stages, thus assisting such process [5]. Thokala and Duenas [6] define four main elements in the MCDM: the criteria by which the alternatives are evaluated, the alternatives to be evaluated, weights of criteria that measure the relative importance of each criterion in comparison with others and scores that reflect the value of the expected performance of the alternatives. MCDM is one of the most well-known branches of decision making [7].

Multiple Criteria Decision Making has been applied in areas of knowledge such as: energy, environment and sustainability, supply chain management, material, quality management, geographic information systems, construction and project management, security and risk management, strategic management, knowledge management, production management, tourism management, among others [8]. It has generally been used in the face of complex, uncertain and conflicting situations [9].

Decision making related to the health area is complex and difficult because it involves multiple factors, options, imperfect information and different order of preferences to those involved [10]. In this area of knowledge, spatial information has been relevant for the decision making by managers. It is of special interest in epidemiological surveillance, Spatial Decision Support Systems (SDSS) which can point out regions by priority level in a geographical region, according to epidemiological measures and specific knowledge about a disease, in order to prevent epidemiological outbreaks.

SDSS has been applied in various areas of knowledge such as flood risk management [11], earthquake disasters [12], infrastructure planning [13] and public education management [14]. SDSS has not been employed in health-related tasks in a significant proportion [15]. SDSSs combine spatial and non-spatial characteristics in the decision making process. Spatial data can be represented by the geographical coordinates of a location and its spatial relationships, being essential in the final decision making process [15]. Ferretti and Montibeller [16] highlighted the relevance of MCDM to the SDSS and the challenges of integrating spatial data and MCDM methods.

In the scientific literature some studies address the spatial relationship with the multiple criteria [16–21]. It was possible to identify Multicriteria Spatial Decision Support Systems (MC-SDSS), an SDSS class based on the association of Geographic Information System (GIS) and MCDM, which uses spatial data and decision maker preferences to provide the final decision [3, 21]. It has been approached in three distinct ways: conventional MCDM for spatial decision making, spatially explicit MCDM and spatial multiobjective optimization [21].

According to Malczewski and Rinner [21], the conventional MCDM approach to spatial problems is usually characterized by not satisfying the fundamental properties of spatial data such as spatial dependence and heterogeneity. Therefore, it assigns spatial homogeneity to the preferences of the decision maker and value functions [21]. Conventional MCDM has been employed to treat spatial problems [21] and the frequently used methods are: weighted linear combination (WLC) and related procedures [22, 23], reference ideal methods [24], the analytical hierarchy and network process [25], and outranking methods [26].

In this paper, we propose using WLC as a combining rule of a set of SDSS, where each one of them analyzes specific aspects of a complex problem. Each SDSS provides a preliminary assessment regarding a specific variable of the problem and its dimension, and it generates georeferenced maps pointing out priority clusters with respect to that variable. In the following, a WLC serve as a combining rule of the previous results, in order to provide a final decision map with respect to levels of priority for the fight against a disease.

To elucidate the proposed approach, tuberculosis (TB) data from the state of Paraíba, Brazil in 2013 were used. Therefore, this work aims to contribute with a new combining rule for spatial decision making using the weighting of criteria derived from spatial epidemiological information. The use of this approach in health surveillance can provide a scientific way of setting priorities for the fight against diseases, such as TB.

## Methods

SDSS has been employed in healthcare as in the following examples. In [27], a system was used to analyze the spatial variation of accessibility to certain services within the area of a city, based on network analysis, and share the results with potential users (citizens and decision makers) in the form of a web application. Another research developed a SDSS and evaluated its usefulness to support management of a program to eliminate malaria and verified high acceptability as an operational data management and surveillance system [28].

In addition, other studies have shown that the system has been successfully used to support malaria eradication in other countries [29], in health care [30] and epidemiological problems, such as: acquired immune deficiency syndrome (AIDS) and dengue fever [31, 32].

Given the applicability of SDSS, the one developed by Moraes et al. [31] stands out for presenting an architecture that considers epidemiological aspects for decision making in public health management. The data are representative for area elements, i.e., the exact geographical location of each occurrence is unknown, but the total occurrence value of each area can be determined. This architecture differs from the others by considering spatial and non-spatial data, inferential statistics, spatial and spatio-temporal analysis agglutinated by a fuzzy rule-based system.

### The architecture of Moraes et al. [31]

**Fig. 1:**
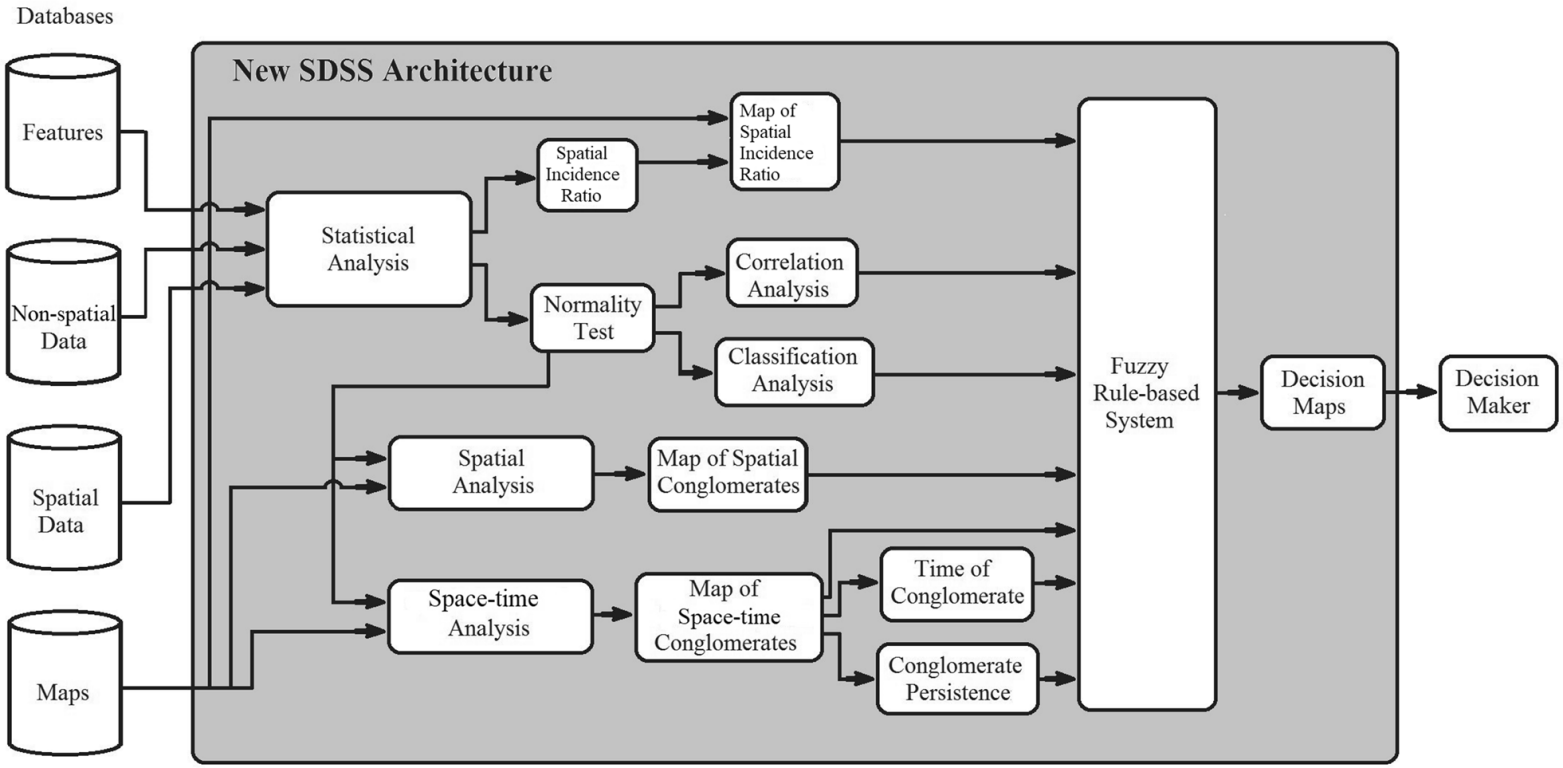
The architecture of Moraes et al. [31]

Moraes et al. [31] proposed an architecture that took into account only one criterion and epidemiological aspects for decision making in public health management. As presented in Fig. 1, this architecture has as inputs a set of attributes, spatial and non-spatial data and maps. It consists of modules of statistical analysis, spatial analysis and space–time analysis, with their respective developments and a fuzzy rule-based system. All modules and the fuzzy rule-based system will be explained below for a better understanding.

In the statistical analysis module, descriptive analyzes, tables and graphs can be generated in order to know the distribution and statistical characteristics of the disease under study. Subsequently, the Spatial Incidence Ratio (SIR) seeks to know the relative contribution of observed cases in an area in relation to the population of such area. For this, it is essential to know some concepts of spatial analysis: geographic region and geo-object. The geographic region is the defined area of the study in which the events of interest occur, and the geo-object is exposed by a set of diverse and geographically identifiable objects within the geographic region [33]. For example, Brazil could be the geographic region under study and each of its states would be a geo-object. Formally, let *A* be a geographic region constituted by a set of *n* geo-objects indicated by *a*_*1*_, *a*_*2*_,…,*a*_*n*_. Let *V*(*a*_*i*_) be a random variable that refers to the number of occurrences of an epidemiological event in a limited period of time within each geo-object in *a*_*i*_, expressed as *v*_*1*_, *v*_*2*_,…,*v*_*n*_. Lastly, let *X*(*a*_*i*_) be the population under risk for that epidemiological event within each geo-object of a *a*_*i*_, denoted by *x*_*1*_, *x*_*2*_,…,*x*_*n*_ [34, 35]. The SIR for each geo-object *a*_*i*_ can be presented as in the following equation [34, 35]:

1 $${\text{SIR}}\left( {{\textit{a}}_{\textit{i}} } \right) \textit{=} \frac{{\frac{{{\textit{V}}\left( {{\textit{a}}_{\textit{i}} } \right)}}{{{\textit{X}}\left( {{\textit{a}}_{\textit{i}} } \right)}}}}{{\frac{{\mathop \sum \nolimits_{{{\textit{i}}\textit{=} \textit{1}}}^{\textit{n}} {\textit{V}}\left( {{\textit{a}}_{\textit{i}} } \right)}}{{\mathop \sum \nolimits_{{{\textit{i}} \textit{=} \textit{1}}}^{\textit{n}} {\textit{X}}\left( {{\textit{a}}_{\textit{i}} } \right)}}}}$$

that is, the SIR of the geo-object *a*_*i*_ is given by the incidence ratios of the occurrence of the event in that geo-object with respect to the geographic region. Table 1 shows the interpretation of the SIR according to its classification.

**Table 1:** Values and interpretation of the Spatial Incidence Ratio

Values	Interpretation
SIR(*a*_*i*_) = 0	When the geo-object under study has no epidemiological incidence
0 < SIR(*a*_*i*_) < 0.5	The SIR is less than half of the total incidence of the geographical region
0.5 ≤ SIR(*a*_*i*_) < 1.0	SIR is more than half of the total incidence, but is less than the epidemiological incidence of the geographical region
1.0 ≤ SIR(*a*_*i*_) < 1.5	Then SIR is higher than the total incidence of the geographical region by less than 50%
1.5 ≤ SIR(*a*_*i*_) < 2.0	The SIR exceeds the global incidence of the geographical region by more than 50%
SIR(*a*_*i*_) ≥ 2.0	Then SIR is two or more times higher than the total incidence of the geographical region

Also in the statistical analysis module, the normality test aims to verify if a dataset can be approximated by the normal distribution [36]. One possible test to use is the Lilliefors test. This will define the set of possible methods to be used later.

Correlation analysis is a measure used to verify the degree of correlation between variables [36]. If the study data do not present a normal distribution, nonparametric statistical tests, such as the Spearman correlation coefficient, can be used. Spearman’s correlation coefficient is a measure of association between two variables and requires both to be measured at least on an ordinal scale [36].

2 $$r_{s} \textit{=} \textit{1} - \frac{{\textit 6\sum \nolimits_{i = 1}^{N} d_{i}^{2} }}{{N^{\textit 3}} - N}$$

where ${d_{i}}^{{2}}$: difference between each rank of the values corresponding to the observations and *N*: number of pairs of observations.

The values of the coefficient range from − 1 to 1, with 0.75 ≤ r_s_ ≤ 1.00 referring to a strong correlation, 0.50 ≤ r_s_ < 0.75 a moderate correlation, r_s_< 0.50 a weak correlation, 0 indicates absence of correlation and r_s _= ± 1 is a perfect correlation.

In the classification analysis module, the fuzzy parallelepiped method can be used to determine the urban areas scattered in a heterogeneous environment, allowing to assign a geo-object to more than one priority level for the fight against diseases, according to a certain degree of pertinence. In general, fuzzy methods have been shown to be more appropriate than conventional methods for the classification of heterogeneous areas [37].

The spatial analysis module is intended to detect and infer spatial clusters. One possible method is the Circular Scan Statistic [38]. This methodology uses a circle, positioned on the center of mass of each geo-object of the geographic region under study, in order to identify the spatial clusters in which the occurrence of the event is significantly more likely inside the circle than outside it. The radius of the circle is increasing and can range from zero to a maximum value of 50% of the population at risk [38]. Due to the nature of the epidemiological data being discrete, the Poisson probabilistic model is a good alternative. In general, a significance level of 5% is used for the hypothesis tests of Monte Carlo simulations with 999 random replications of the data with the null hypothesis of spatial randomness [38].

According to the assumption of the Poisson model, the radius with values of *p*(*a*) and *q*(*a*) that maximize the likelihood function conditioned to the total of observed cases are computed, where the likelihood ratio is understood as the one that tests the hypothesis of an event occurring randomly. Assuming a possible cluster, the Scan (*S*) statistic can be calculated according to the following equations [38]:

3 $${\textit{S}} = {{\textit{max}}}_{{{\textit{a}}\in{\textit{A}}}} \frac{{{\textit{L}}\left( {{\textit{a}},{\hat{\textit{p}}}\left( {\textit{a}} \right),{\hat{\textit{q}}}\left( {\textit{a}} \right)} \right)}}{{{\textit{L}}_\textit{0}}}$$

where *A* represents a geographic region formed by a set of *n* geo-objects, or of all possible cluster candidates, denoted by: *a*_*1*_, *a*_*2*_,…, *a*_*n*_, with ${\hat{\textit{p}}}\left( {\textit{a}} \right)$ and ${\hat{\textit{q}}}\left( {\textit{a}} \right)$ being estimates of ${\textit{p}}\left( {\textit{a}} \right)$ and ${\textit{q}}\left( {\textit{a}} \right)$, where *p*(*a*) is a probability of individuals being inside the circle, while *q*(*a*) is a probability of individuals being outside. *L*_*0*_ can be defined as:

4 $${\textit{L}}_\textit{0} \textit{=} \frac{{{\textit{O}}^{\textit{O}} \left( {{\textit{X}} - {\textit{O}}} \right)^{{{\textit{X}} - {\textit{O}}}} }}{{{\textit{X}}^{\textit{X}} }}$$

where *O* is the total number of observed cases across the entire geographic region *A* and *X* is the total population exposed to risk in the geographic region *A*. ${\textit{L}}\left( {{\textit{a}},\;{\hat{\textit{p}}}\left( {\textit{a}} \right),\;{\hat{\textit{q}}}\left( {\textit{a}} \right)} \right)$ can be defined as:

5 $${\textit{L}}\left( {{\textit{a}},\;{\hat{\textit{p}}}\left( {\textit{a}} \right),\;{\hat{\textit{q}}}\left( {\textit{a}} \right)} \right) \textit{=} \frac{{\textit{exp} \left[ { - {\textit{p}}\left( {\textit{a}} \right){\textit{X}}\left( {\textit{a}} \right) - {\textit{q}}\left( {\textit{a}} \right)\left( {{\textit{X}}\left( {\textit{A}} \right) - {\textit{X}}\left( {\textit{a}} \right)} \right)} \right]}}{{{\textit{O}}\textit!}} {\textit{p}}\left( {\textit{a}} \right)^{c\left( a \right)} {\textit{q}}\left( {\textit{a}} \right)^{{{\textit{C}} - {\textit{c}}\left( {\textit{a}} \right) }} \prod_{\textit{i}} {\textit{c}}\left( {\textit{i}} \right)$$

where *exp* represents the exponential function, *c*(*a*) and *c*(*i*) (*a*, *i* *=* *1,* *2,…,n*) are, respectively, the number of cases in the geo-object *a* and in the geo-object *i* and *X*(*a*) is the number of individuals at risk in geo-object *a*.

In the space–time analysis, we try to detect clusters that happen in space and time concomitantly. One possible methodology is the space–time Scan statistic. The main difference between the Scan circular statistic and the space–time Scan is the time period and the cylindrical scanning format. The sweep is made by means of cylinders that present a circular base, equivalent to the geographic dimension, and the height, corresponding to the interval of time. This base is centered on one of the centroids of the geo-objects contained in the geographic region of study with the radius varying in size continuously. It is indicated that the time interval is limited to half of the total period and the geographical dimension to half the number of expected cases [39]. Therefore, the cylindrical window moves in space and time so that for every possible geographic location, it also visits every possible period of time, translating to overlapping cylinders of different sizes that are tested for the probability of composing a space–time cluster. The significance of a cluster is calculated using the Monte Carlo simulation, of which the null hypothesis asserts its non-existence and the alternative hypothesis that there is at least one cluster with a 5% level of significance [39].

In space–time Scan, time can be approached as a retrospective or prospective analysis. Retrospective analysis aims to detect clusters over a given period of time by performing a single analysis [39], while in the prospective it happens repeatedly in the period of time [40].

The results from these modules serve as input to a fuzzy rule-based system which agglutinates this information and produces as output a map indicating areas with different levels of priorities for the fight against diseases. In the study, a fuzzy rule-based system based in [41] was used. The knowledge used in the rule base comes from experts in the specific field of application. In this case, the rules come from the relationships between the epidemiological, spatial and spatiotemporal statistics of the disease and the priority levels that must be given to combat them.

The fuzzy set was proposed by [42] and is characterized by pertinence functions, assigned to each object of the set, which vary between zero and one. Let H be a space of points, with a generic element of H denoted by h. A fuzzy set B in H is characterized by a pertinence function μ_B_ (h) that assigns to each point in H a real number in the interval [0,1], where μ_B_ (h) corresponds to the pertinence degree of h in B. A fuzzy rule-based system is composed by: fuzzification, rules, inference and defuzzification [43, 44]. Fuzzification has the intent of transforming a non-fuzzy set in a fuzzy set. The rules are formulated with linguistic variables that are represented by a variable of which the values are words or phrases in a natural or artificial language. In the inference process, logical connectives were used with the objective of indicating the fuzzy relationship that models the rules, while the defuzzification corresponds to the last stage, in which the resulting fuzzy set is converted to a numeric value [43, 44].

The modules explained above can be suppressed or modified in their methodology, according to the needs of the problem in question [30]. It allows an adaptive contribution in the process of decision making.

### WLC for spatial decision making

The WLC is a simple, easy-to-understand method and has been consistently used in the MCDM method with GIS [23]. It can be defined as a technique which uses spatial data and decision maker preferences to provide the decision map [21]. The WLC takes into account two components: the weight for each criterion and the value function. The weight represents the importance of each criterion in the understanding of the decision maker (expert). The sum of the weights must be equal to one [23, 45]. The value function converts the different levels of a criterion on a comparable scale [45]. The WLC method to choose the best alternative for each geo-object can be expressed by the equation:

6 $${\textit{P*}}\left( {\textit{a}} \right) \textit{=} {\textit{max}}_{{\textit{i}}} \left\{ {\sum\limits_{{{\textit{j}} = 1}}^{{\textit{K}}} {{\textit{z}}_{{{\textit{ij}}}} } \left( a \right){\textit{w}}_{{\textit{j}}} } \right\},\forall {\textit{i}} \textit{=} \textit{1,}\textit{2,}{\textit{...,m}}{\textit{;}}\quad {\textit{j}} \textit{=}\textit{1,}\textit{2,} {\textit{...,K}}\quad{and} \quad \textit{a} \textit{=} \textit{1,}\textit{2,}{\textit{...,n}}$$

where P*(*a*) is the best score among the *m* alternatives for each geo-object *a*; *z*_*ij*_ is the value function of the *i*-th alternative in terms of the *j*-th criterion for each geo-object, and *w*_*j*_ is the weight attributed to each of the *K* criteria.

### The new approach

**Fig. 2:**
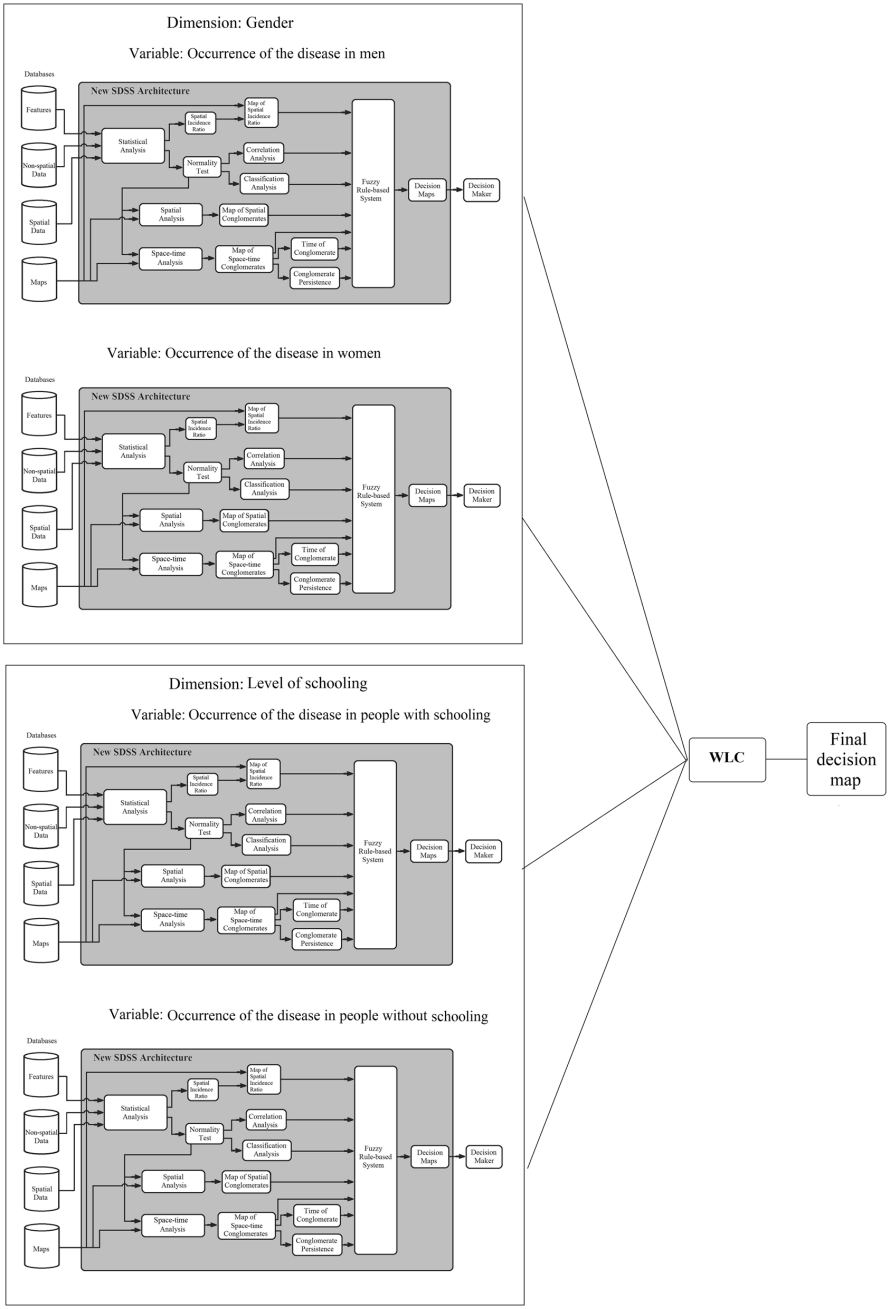
The new approach

In this paper, we propose using and replying the architecture proposed by Moraes et al. [31] for each variables that composes the dimensions of a spatial problem, generating georeferenced maps for each of them. Subsequently, the WLC is applied as a combining rule for each geo-object of the geographic region (Fig. 2). For example, the TB can be analyzed according to several georeferenced dimensions that are analyzed separately, according to their relevant variables. For example, the dimension “gender” has two epidemiological variables: “occurrence of the disease in men” and “occurrence of the disease in women”; the dimension “level of schooling” has two epidemiological variables: “occurrence of the disease in people with schooling” and “occurrence of the disease in people without schooling”. It is noteworthy that the variables are grouped in these dimensions due to the similarity of knowledge used in the rule-based system. Experts in the specific field of application in line with the scientific literature developed the rules for each SDSS and assigned the weights of each criterion. Specifically, each geo-object of the geographic region is processed, according to its own spatial information, by an SDSS using spatial and non-spatial data, inferential statistics and spatial and spatio-temporal analysis, which are then grouped together by a fuzzy rule-based system that will produce a georeferenced map. The results from each variable are combined by the WLC as a criterion in a MCDM problem, producing a final decision map about the priority levels for fight against a disease.

## Results

### Application of the new approach

Tuberculosis is an infectious disease of chronic evolution, being one of the ten leading causes of death worldwide. In 2017, it is estimated that 10 million people throughout the world developed the disease, with approximately 5.8 million being males, 3.2 million females and 1.0 million children. In the same year, about 1.3 million deaths were registered [46]. According to the new classification of the World Health Organization (2016–2020), Brazil ranks 20th in the list of 30 countries with high TB burden and 19th in the list of 30 countries with high tuberculosis–human immunodeficiency virus (HIV) co-infection [46]. In view of the seriousness of this epidemiological scenario, the new approach was applied on TB notified cases in the city of João Pessoa, in the Brazilian state of Paraíba, in order to demonstrate its usefulness.

A total of 2352 cases of TB were reported in the city of João Pessoa between 2009 and 2013. The dimensions used in the study were gender (occurrence of the disease in men and occurrence of the disease in women) and level of schooling (occurrence of the disease in people with schooling and occurrence of the disease in people without schooling). Each of the dimensions is analyzed initially by the architecture proposed by [31] independently, producing as a result a map for that variable. The resulting of each variable, in turn, became input criteria to the MCDM according to its specific considerations, composing the new approach proposed in this article (Fig. 2).

Weights for each criterion were assigned by specialists in the specific field of application, who were also based on the disease-specific scientific literature. These, in turn, can be modified according to certain particularities. Accordingly, the rules that make up each rule-based fuzzy system within each SDSS should also be changed. Studies show that TB is more frequent in males individuals [46–49], perhaps as a result of men being more prone to alcoholism, malnourishment or co-infection with the HIV virus [50]. Regarding the educational level, investigations show association of TB with illiteracy and low level of schooling [50, 51]. Low level of schooling is associated with delayed diagnosis of the disease [52], and adhesion to treatment [53]. Illiteracy is also associated with TB mortality [54]. Thus, the highest weights were attributed to male individuals and those with no schooling.

$${\textit{w}}_{\textit{j}} = \left[ {0.30\quad 0.15\quad 0.40\quad 0.15} \right]$$

where 0.30: weight attributed to the male individuals; 0.15: weight attributed to the female individuals; 0.40: weight attributed to those with schooling; 0.15: weight attributed to those without schooling.

**Fig. 3:**
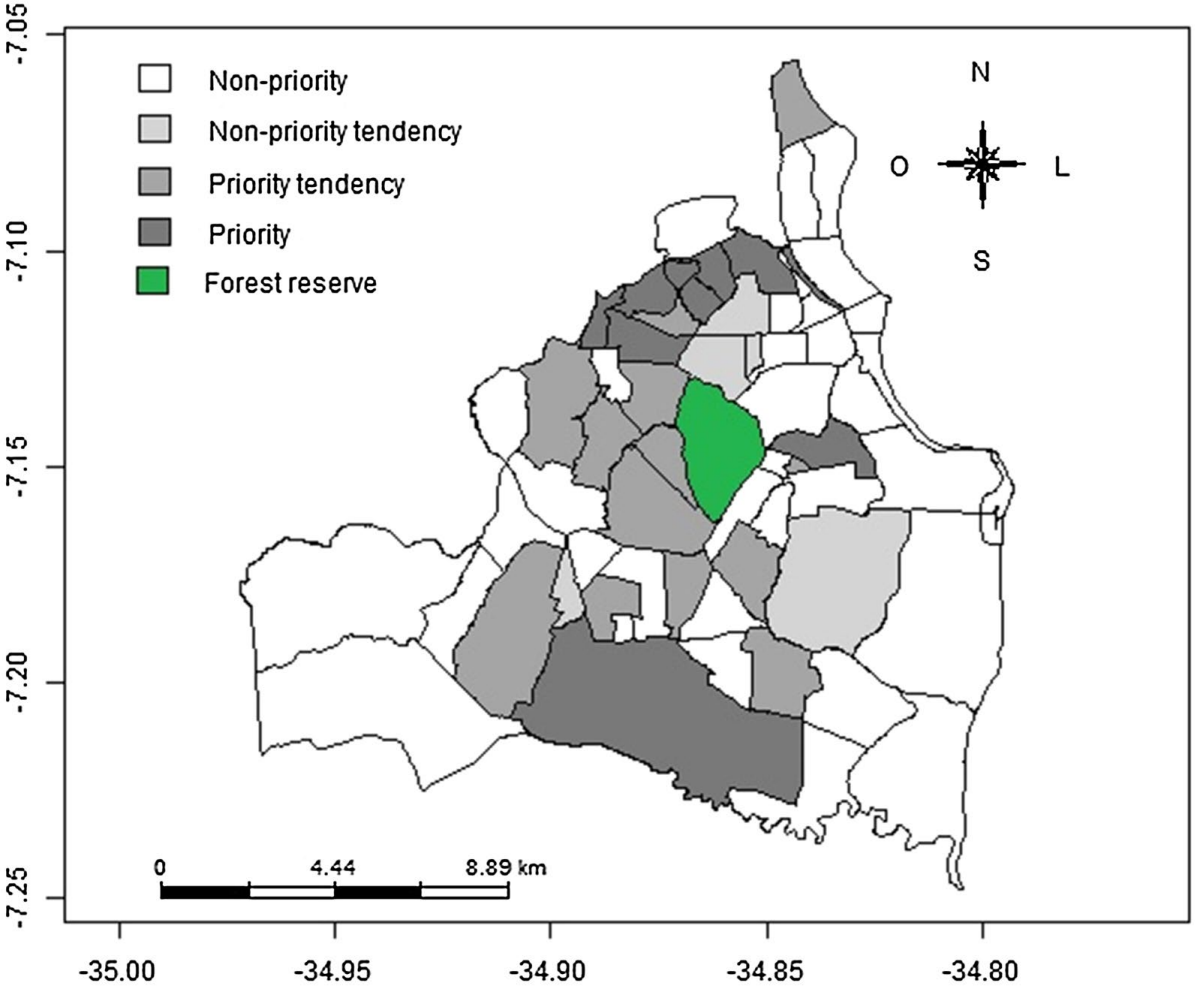
Map resulting from the application of the new approach in cases of tuberculosis the city of João Pessoa, Paraíba, Brazil, for the year 2013

As a final result, a new map was obtained showing the four alternatives for the fight against diseases: “non-priority”, “non-priority tendency”, “priority tendency” and “priority”, i.e., priority levels for each geo-object present in the geographic region being studied. The “non-priority tendency” applies in the case of a neighborhood that does not belong to a significant spatial cluster, but has had negative correlation analysis of the SIR in the last 3 years. The “priority tendency” refers to neighborhoods that do not belong to a significant spatial cluster, but present positive correlation analysis of the SIR. This map refers to the city of João Pessoa, representing the four priority levels for the fight against TB in each neighborhood. Figure 3 presents the 36, of the 64 neighborhoods of the city, which were considered to be “non-priority”. They were dispersed throughout the municipality. Thirteen presented “priority tendency”, with a greater concentration in the west and ten were considered “priority” for the fight against TB. Five presented a “non-priority tendency”, of which the majority were located in the northwest region of the city.

## Discussion

From the epidemiological point of view, the alternatives “priority” and “priority tendency” require immediate and future interventions by the public manager, respectively. These alternatives help the manager to make a decision in a coherent and assertive way. In addition, if there is availability and resources, this intervention can be done immediately in both situations.

Most of the neighborhoods that were considered “priority” or “tendency priority” have higher population densities or socioeconomic vulnerability. In the region with the highest concentration of priority neighborhoods there is a prison, in addition to some points of prostitution. The prevalence of TB is higher in the prison population, which can be justified by overcrowding and poor lighting and ventilation conditions [55].

The research of [56] stated that the spatial distribution of TB was more concentrated in neighborhoods with higher population and intradomiciliary densities, corroborating the results of the present study. Another study found that TB occurred predominantly in the central region of Divinópolis, Minas Gerais, Brazil, and a significant association can be found between the disease and the sites with the highest population density [49], similarly to the findings of this work. In a study conducted in Fortaleza, Brazil, it was found that TB cases were agglomerated in areas with high informal settlement rates [57].

In general, TB is a disease that affects the economically disadvantaged population [58]. The occurrence of TB is associated to socioeconomic inequalities [56]. As such, it is important to articulate several public services, such as the health, housing, infrastructure, social assistance and education sectors, with the objective of minimizing the social burden of TB [56].

Using the architecture proposed by [31] through replication for each variable of the problem, an in-depth analysis of each one was possible. They composed the set of criteria in the context of the final decision making for each geo-object of the geographic region. Therefore, this approach can contribute to the management of epidemiological surveillance taking into account the administrative and epidemiological information, especially in what concerns the priority areas for the fight against diseases. Another contribution of this work is a new combination rule for spatial decision making using the weighting of criteria derived from spatial epidemiological information. As epidemiological problems of this nature are all structured in a similar way, it is possible to use this new approach for analyzing different diseases. It is worth noting that this approach is general and can be applied to other problems in health sciences, as well as in other areas beyond that, taking into account georeferenced information.

The limitation of this research refers to the use of secondary data, which requires information of good quality and accurately recorded, and such information is sometimes not available. However, future works may increase the number of epidemiological or surveillance information.

## Conclusion

The present study presented an innovative approach with an interdisciplinary point of view, involving statistical and spatial analysis, multicriteria decision making and epidemiology. No other similar approach was found in the scientific literature. It allowed the application of epidemiological data and the identification of areas with different levels of priority for the fight against diseases. This approach can be adopted for other diseases, using specific modules according to the problematic in question. It allows an adaptive contribution in the process of decision making using georeferenced data.

## Data Availability

Not applicable.
